# Correspondence

**Published:** 1963-10

**Authors:** Lothe Sahlmann


### CORRESPONDENCE

*The Editor, Bristol Medico-Chirurgical Journal*

Dear Sir,

Dr. MacLachlan's Presidential Address on Rudolf Steiner's life and work, printed in the January 1963 issue of the Journal will need a good amount of correction, as many misrepresentations and misunderstandings are contained in it. I should be grateful if you would publish this short letter in which I will try to refute some of these mistakes.

An actual approach to Rudolf Steiner's wide and very deep-reaching conception of man's relationship to himself and to the world, can only be acquired by intense study of the fundamental books and lectures. Unless such a basic knowledge has been gained, any applied activities and practical outcomes in the field of education, medicine, biology, mathematics etc. cannot be understood.

> *Page* 7, *III line* 6*:* "The same background which produced Adolf Hitler produced Dr. Rudolf Steiner."

> *Page* 8, *line* 19*:* "Just as Hitler sought a political explanation . . . so Steiner sought a scientific and religious one."

Rather absurdly Dr. MacLachlan throws together two personalities into one statement, though needless to say their motives and striving were as diametrically opposed as possible; their age differed by more than one generation. Rudolf Steiner was a citizen of the 19th century. He was bred and reared on the philosophical and scientific development of that period and had written his main books by the beginning of the first world war. After the war he tried to bring about the Threefold Commonwealth for Central Europe which would have given political independence to the various national groups, liberty of conscience, and spiritual freedom to the individual, as well as a far-sighted economic co-operation through the establishment of Economic Associations. This idea was brought to naught by the historic developments.

> *Page* 8, *line* 2*:* "Following years of study at Weimar and Berlin, Steiner took to lecturing on what he claimed to be "spiritual science" as opposed to "natural science" and based on the work of Goethe, . . ."

In 1889 Rudolf Steiner was called to Weimar to edit the scientific works of Goethe. He himself did not base his work on Goethe though he admired him greatly and appreciated his way of approaching the world of the living, growing organism. Our thinking can only grasp the laws of physics and chemistry, the laws of the lifeless. To follow up the laws of growth, reproduction and metamorphosis, that is, of the living organism, we have to bring our thoughts into inner activity. The element of time appears in the process of thinking. Goethe, in his "Metamorphosis of Plants" has shown how to approach the problem of life. This, not the actual observational results, is his great contribution towards a new science. Steiner has recognized this fully, but has gone further.

> *Page* 8, *line* 6*:* "Just before the outbrook of the First World War, Steiner broke with the Theosophical Movement which was not Western enough in its conceptions of spiritual phenomena for his Teutonic mind;"

> *Page* 9, *line* 25*:* "Dismissed by Annie Besant from the Theosophical Society, his Spiritual Science was not to the liking of the Germans licking their wounds after the 1st World War."

Steiner severed his connection with the Theosophical Society in 1913 because of their fundamental opposition to Christianity. At the same time he began to build the first Goetheanum in Switzerland and from then on throughout the war-years, and after directed his activities from Dornach.

> *Page* 8, *line* 20*:* "It all began, said Steiner, with Isaac Newton and the birth of Natural Science."

It is by no means clear what Dr. MacLachlan understands by "It all began." Rudolf Steiner was most anxious to give a clear and comprehensive exposition of the development of Man's Consciousness throughout the ages, and did so from very many different points of view in many expositions. The 15th Century was a decisive turning-point in human history, as Man began to enquire into the world around him, developing, in ever increasing precision, means and ways to enhance the acuteness of his senses. As a necessary consequence he cut himself off from a more spiritual insight, which man of older times still possessed. This was an unavoidable step, in order to develop to the fullest his own personality and awareness of his Ego. However, the more science progressed, the greater became the abyss between human thinking and morality. There was and still there is no inner link connecting the two. The consequences thereof can be vividly felt in the various social, political and moral problems our world is facing to-day. Steiner has shown how to bridge the gap which has arisen. In his "Philosophy of Spiritual Activity" (Philosophie der Freiheit) he wrestles with the fundamentals of human consciousness and powers of thinking and their interaction with the phenomena of morality and volition. What is given in a philosophical form in this book, he later on approaches and widens from the evolutionary, historical, biological, and ethical point of view.

> *Page* 9, *2nd par.:* "After his death however, this philosophical curiosity was taken up by others both on the Continent and in this country; for, though Steiner had a technical and not a classical education, the colossal arrogance of the system extended to every sphere of life—to politics, education, medicine, agriculture and architecture."

Certainly, for the specialist of to-day with his detailed, though necessarily onesided knowledge of his particular subject, a universal mind like Steiner's must appear fantastic. However, could one not imagine that a fundamental re-appraisal of Man's own true existence and relation to the world could give a more universal key with which to approach in a new light the various fields of our existence?

> *Page* 9, *line* 34*:* ". . . anthroposophy remains in this country as a specialized system of education for handicapped children, and there are still those who hold Steiner's curious views on physiology and the treatment and cure of the sick."

There is also in this country a number of schools for normal children run on the methods and principles laid down by Rudolf Steiner. The education of the handicapped child takes a special place and has in many a way proved to be pioneering. It is based on a description of Man which, of course, includes all the up-to-date scientific knowledge, but goes further, adding many fundamental concepts as to Man's physical, psychological, and spiritiual make-up and their exact interaction. One of these contributions, mentioned in a superficial manner by Dr. MacLachlan, is the interconnection of bodily processes and psychological functions, as described by Rudolf Steiner for the first time in 1917 (Von Seelenrätseln) He points out how our soul-activities of thinking, feeling and willing are coordinated each in an entirely different way to our organic functions. The body as an entirety, not only the nervous system is the physiological carrier of the soul life.

Our perception and power of cognition are based on the sensory-nerve organization and depend entirely on its integrity. Though the brain is the tool for the thought-activity, it is by no means the producer of it. Only in our thinking are we awake and fully conscious, we can penetrate it with utter lucidity and absolute clarity. Our volition is connected to all those activities which make up our metabolic processes, consisting of the anabolic and catabolic changes which continuously happen within our organs, glands, muscles etc. This life of will remains in the unconscious. We only intend to carry out a willed movement; we have a concept of the intention and perceive the result, the actual happenings however in the blood capillaries, the chemical interchanges in the muscles, the act of contraction and flexion of an arm, all this remains in darkness. Nor can we possibly have a direct knowledge of the digestive processes; if we do so, we are ill (pain, organ hallucinations). With regard to our willing we are permanently in a state of unconsciousness, we are actually as if in sleep.

In between the life of thought and will there stands the feeling, swaying continuously towards the one or the other realm, harmonizing and balancing the other soul-activities. Physiologically, our emotional existence is based on the rhythmical processes of breathing and circulation.

It is obvious that the three soul forces, thinking, feeling and willing, hardly ever occur as an isolated factor. In the small child they interact chaotically and only gradually do we isolate them from each other; but never entirely, unless we become ill. Such a concept is fundamental, it leads to a new approach towards an understanding of the human will in all its physiological, psychological and moral implications. Man's soul-life is not only a function of the Nervous System, but feeling and willing are coordinated *directly* to rhythmical, respectively metabolic processes; the nerve is only the mediator to bring them into consciousness. Obviously, such an outlook also alters any approach to illness; and possible therapeutic measures. It also offers, amongst others, very definite views on the constitution of the human being a very fruitful background to the study, diagnosis and treatment of the abnormally constituted child.

As to Dr. MacLachlan's description of a case history and treatment of a patient with generalized tubercular lesions one would have to go very fundamentally into a more specialized study. He quotes terms, the meaning of which he has not at all understood, stigmatizing them as "jargon".

Perhaps by finding out their significance, the role they play as essential qualities and processes in man's make-up he would see the problem in a different light. Naturally they must be sheer names and empty phrases, as long as deeper comprehension, study and contemplation has not been given to an approach to man, as Steiner has indicated: An approach which takes into full account what Science to-day has to give, but extends it to man's threefold relationship, to his bodily nature, to his mind, and to the World of the Spirit.

Yours faithfully,

LOTHE SAHLMANN.

